# 5,8-Dimethyl-3-methyl­ene-2-oxo-3,3a,4,5,5a,6,8a,8b-octa­hydro-2*H*-1-oxa-s-indacene-5-carbaldehyde

**DOI:** 10.1107/S1600536811018344

**Published:** 2011-05-20

**Authors:** Mohamed Moumou, Ahmed Benharref, Moha Berraho, Jean-Claude Daran, Mohamed Akssira, Ahmed Elhakmaoui

**Affiliations:** a Laboratoire de Chimie Bioorganique et Analytique, URAC 22, BP 146, FSTM, Université Hassan II, Mohammedia-Casablanca 20810 Mohammedia, Morocco; b Laboratoire de Chimie des Substances Naturelles, URAC16, Faculté des Sciences Semlalia, BP 2390 Bd My Abdellah, 40000 Marrakech, Morocco; c Laboratoire de Chimie de Coordination, 205 route de Narbonne, 31077 Toulouse Cedex 04, France

## Abstract

The title compound, C_15_H_18_O_3_, was synthesized from 9α-hy­droxy­parthenolide (9α-hy­droxy-4,8-dimethyl-12-methyl­ene-3,14-dioxatricyclo­[9.3.0.0^2,4^]tetra­dec-7-en-13-one), which was isolated from the chloro­form extract of the aerial parts of *Anvillea radiata*. The five-membered lactone ring has a twisted conformation, while the six- and five-membered rings display chair and envelope conformations, respectively. The dihedral angle between the two five-membered rings is 50.57 (11)°.

## Related literature

For the isolation and biological activity of 9α-hy­droxy­parthenolide, see: Abdel Sattar *et al.* (1996 ▶); El Hassany *et al.* (2004 ▶). For the reactivity of this sesquiterpene, see: Castaneda-Acosta *et al.* (1993 ▶); Neukirch *et al.* (2003 ▶); Der-Ren *et al.* (2006 ▶); Neelakantan *et al.* (2009 ▶). For conformational analysis, see: Cremer & Pople (1975 ▶).
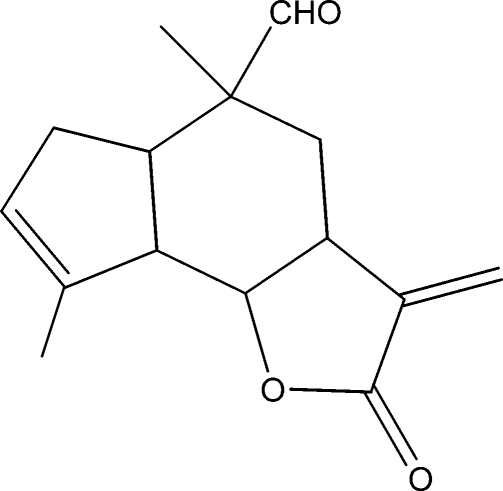


## Experimental

### 

#### Crystal data

C_15_H_18_O_3_
*M* *_r_* = 246.29
Orthorhombic, 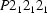

*a* = 9.5293 (3) Å
*b* = 9.7885 (3) Å
*c* = 13.7524 (4) Å
*V* = 1282.79 (7) Å^3^
*Z* = 4
Mo *K*α radiation
μ = 0.09 mm^−1^
*T* = 173 K
0.50 × 0.33 × 0.08 mm

#### Data collection

Bruker APEXII CCD area-detector diffractometer
22932 measured reflections
1517 independent reflections
1403 reflections with *I* > 2σ(*I*)
*R*_int_ = 0.026

#### Refinement

*R*[*F* ^2^ > 2σ(*F* ^2^)] = 0.031
*wR*(*F* ^2^) = 0.085
*S* = 1.09
1517 reflections
165 parameters
H-atom parameters constrained
Δρ_max_ = 0.18 e Å^−3^
Δρ_min_ = −0.16 e Å^−3^

### 

Data collection: *APEX2* (Bruker, 2005 ▶); cell refinement: *APEX2* and *SAINT* (Bruker, 2005 ▶); data reduction: *SAINT*; program(s) used to solve structure: *SHELXS97* (Sheldrick, 2008 ▶); program(s) used to refine structure: *SHELXL97* (Sheldrick, 2008 ▶); molecular graphics: *ORTEP-3 for Windows* (Farrugia, 1997 ▶); software used to prepare material for publication: *WinGX* (Farrugia, 1999 ▶).

## Supplementary Material

Crystal structure: contains datablocks I, global. DOI: 10.1107/S1600536811018344/sj5146sup1.cif

Structure factors: contains datablocks I. DOI: 10.1107/S1600536811018344/sj5146Isup2.hkl

Additional supplementary materials:  crystallographic information; 3D view; checkCIF report

## Figures and Tables

**Fig. 1.**
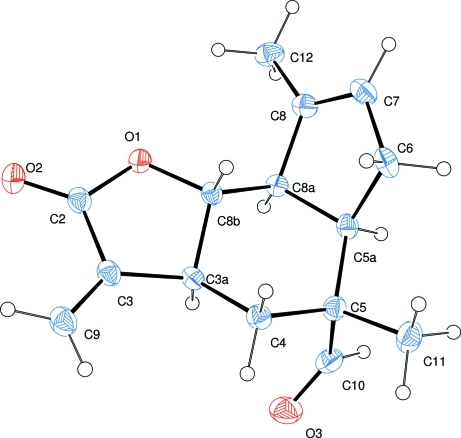
: Molecular structure of the title compound with the atom-labelling scheme. Displacement ellipsoids are drawn at the 30% probability level. H atoms are represented as small spheres of arbitrary radii.

## supplementary crystallographic information

### Comment

The natural sesquiterpene lactone (9α - hydroxypartenolide) is the main constituent of the chloroform extract of aerial parts of Anvillea radiata (El Hassany *et al.*, 2004) and of Anvillea garcini (Abdel Sattar *et al.*, 1996). The reactivity of this sesquiterpene lactone and its derivatives has been the subject of several studies (Castaneda-Acosta *et al.* 1993; Neukirch *et al.*, 2003; Der-Ren *et al.*, 2006; Neelakantan *et al.*, 2009), in order to prepare products with high added value for use in industrial pharmacology. In the same context, we have treated the 9α-hydroxyparthenolide with boron trifluoride etherate and obtained the 5,8-diméthyl-3-methylen-2-oxo-3,3a,4,5,5a,6,8a,8b- octahydro-2*H*-1-oxa-as-indacene-5-carbaldehyde 64% yield. The structure of this new sesquiterpene derivative of 9α - hydroxypartenolide was determined by ^1^H and ^13^C NMR spectral analysis and mass spectrometry, and was confirmed by its single crystal X-ray structure. The molecule contains three fused rings which exhibit different conformations. The molecular structure of (I), Fig.1, shows the lactone ring to adopt a twisted conformation, as indicated by Cremer & Pople (1975) puckering parameters Q = 0.3329 (18) Å and φ = 304.4 (3)°. The five-membered ring displays an envelope conformation with Q = 0.340 (2)Å and φ = 356.8 (3)°, while the six-membered ring has a chair conformation with QT = 0.5707 (18) Å, θ = 16.39 (18)°, φ = 333.5 (7)°.

### Experimental

Boron trifluoride etherate (1 ml, freshly distilled under reduced pressure) was added *via* syringe over a 10 minute period to a stirred solution of 500 mg (1.89 mmol) of the 9α-hydroxyparthenolide in anhydrous benzene (20 ml), cooled in an ice-bath and maintained under a N_2_ atmosphere. The ice-bath was then removed and stirring was continued for 2 h during which time the solution became cloudy and reddish in colour. The reaction mixture was poured into cooled water and dichloromethane. After shaking, the layers were separated; the organic layer was treated three time with saturated sodium bicarbonate (3x30ml), dried over sodium sulfate and concentrated under reduced pressure. Chromatography of the residue obtained on silica gel with hexane/ ethyl acetate (85/15) as eluent allowed us to isolate in pure 300 mg (1.21 mmol) of 5,8-dimethyl-3-methylene - 2-oxo-3,3a,4,5,5a,6,8a,8 b –octahydro-2*H*-1-oxa-as-indacene-5-carbaldehyde. The title compound was recrystallized from ethyl acetate.

### Refinement

All H atoms were fixed geometrically and treated as riding with C—H = 0.96 Å (methyl), 0.97 Å (methylene), 0.98Å (methine) with *U*_iso_(H) = 1.2Ueq (methylene, methine and OH) or *U*_iso_(H) = 1.5Ueq(methyl). In the absence of significant anomalous scattering, the absolute configuration could not be reliably determined and thus 1100 Friedel pairs were merged and any references to the Flack parameter were removed.

### Crystal data

**Table:**

C_15_H_18_O_3_	*F*(000) = 528
*M**_r_* = 246.29	*D*_x_ = 1.275 Mg m^−^^3^
Orthorhombic, *P*2_1_2_1_2_1_	Mo *K*α radiation, λ = 0.71073 Å
Hall symbol: P 2ac 2ab	Cell parameters from 22932 reflections
*a* = 9.5293 (3) Å	θ = 2.6–26.4°
*b* = 9.7885 (3) Å	µ = 0.09 mm^−^^1^
*c* = 13.7524 (4) Å	*T* = 173 K
*V* = 1282.79 (7) Å^3^	Platelet, colourless
*Z* = 4	0.50 × 0.33 × 0.08 mm

### Data collection

**Table:**

Bruker APEXII CCD area-detector diffractometer	1403 reflections with *I* > 2σ(*I*)
Radiation source: fine-focus sealed tube	*R*_int_ = 0.026
graphite	θ_max_ = 26.4°, θ_min_ = 2.6°
φ and ω scans	*h* = −9→11
22932 measured reflections	*k* = −12→12
1517 independent reflections	*l* = −17→17

### Refinement

**Table:**

Refinement on *F*^2^	Primary atom site location: structure-invariant direct methods
Least-squares matrix: full	Secondary atom site location: difference Fourier map
*R*[*F*^2^ > 2σ(*F*^2^)] = 0.031	Hydrogen site location: inferred from neighbouring sites
*wR*(*F*^2^) = 0.085	H-atom parameters constrained
*S* = 1.09	*w* = 1/[σ^2^(*F*_o_^2^) + (0.0511*P*)^2^ + 0.1837*P*] where *P* = (*F*_o_^2^ + 2*F*_c_^2^)/3
1517 reflections	(Δ/σ)_max_ < 0.001
165 parameters	Δρ_max_ = 0.18 e Å^−^^3^
0 restraints	Δρ_min_ = −0.16 e Å^−^^3^

### Special details

**Table:**

Geometry. All s.u.'s (except the s.u. in the dihedral angle between two l.s. planes) are estimated using the full covariance matrix. The cell s.u.'s are taken into account individually in the estimation of s.u.'s in distances, angles and torsion angles; correlations between s.u.'s in cell parameters are only used when they are defined by crystal symmetry. An approximate (isotropic) treatment of cell s.u.'s is used for estimating s.u.'s involving l.s. planes.

### Fractional atomic coordinates and isotropic or equivalent isotropic displacement parameters (Å^2^)

**Table:**

	*x*	*y*	*z*	*U*_iso_*/*U*_eq_	
C2	−0.05168 (19)	0.3323 (2)	0.83535 (13)	0.0323 (4)	
C3	−0.01615 (19)	0.47821 (19)	0.85677 (12)	0.0307 (4)	
C3A	0.12071 (17)	0.47296 (17)	0.91013 (12)	0.0267 (4)	
H3	0.0996	0.4573	0.9790	0.032*	
C4	0.22933 (18)	0.58577 (18)	0.90494 (13)	0.0301 (4)	
H4A	0.1903	0.6701	0.9303	0.036*	
H4B	0.2568	0.6010	0.8379	0.036*	
C5	0.3576 (2)	0.54344 (18)	0.96536 (13)	0.0311 (4)	
C5A	0.40893 (18)	0.39541 (18)	0.94628 (13)	0.0288 (4)	
H5A	0.4649	0.3698	1.0032	0.035*	
C6	0.5034 (2)	0.3693 (2)	0.85704 (15)	0.0382 (5)	
H6A	0.4700	0.4183	0.8003	0.046*	
H6B	0.6000	0.3950	0.8699	0.046*	
C7	0.4886 (2)	0.2173 (2)	0.84457 (14)	0.0377 (4)	
H7	0.5514	0.1638	0.8095	0.045*	
C8	0.3756 (2)	0.16819 (18)	0.88923 (12)	0.0321 (4)	
C8A	0.29432 (17)	0.28351 (16)	0.93605 (11)	0.0254 (3)	
H8A	0.2558	0.2568	0.9993	0.030*	
C8B	0.18260 (17)	0.34167 (17)	0.86986 (12)	0.0252 (3)	
H8B	0.2253	0.3615	0.8066	0.030*	
C9	−0.0975 (2)	0.5818 (2)	0.83263 (14)	0.0386 (4)	
H9A	−0.1819	0.5660	0.8006	0.046*	
H9B	−0.0705	0.6706	0.8477	0.046*	
C10	0.3226 (2)	0.55169 (19)	1.07397 (14)	0.0379 (4)	
H10	0.3857	0.5104	1.1164	0.046*	
C11	0.4789 (2)	0.6450 (2)	0.95088 (17)	0.0439 (5)	
H11A	0.4487	0.7350	0.9691	0.066*	
H11B	0.5069	0.6451	0.8838	0.066*	
H11C	0.5570	0.6184	0.9907	0.066*	
C12	0.3260 (2)	0.02303 (19)	0.89327 (14)	0.0401 (5)	
H12A	0.3250	−0.0075	0.9596	0.060*	
H12B	0.3882	−0.0337	0.8560	0.060*	
H12C	0.2330	0.0173	0.8668	0.060*	
O1	0.06185 (12)	0.25263 (13)	0.85447 (9)	0.0302 (3)	
O2	−0.16069 (14)	0.28443 (16)	0.80758 (11)	0.0455 (4)	
O3	0.22305 (18)	0.60552 (17)	1.10965 (11)	0.0531 (4)	

### Atomic displacement parameters (Å^2^)

**Table:**

	*U*^11^	*U*^22^	*U*^33^	*U*^12^	*U*^13^	*U*^23^
C2	0.0276 (9)	0.0415 (10)	0.0279 (8)	0.0015 (8)	0.0021 (7)	−0.0003 (8)
C3	0.0266 (8)	0.0389 (9)	0.0266 (8)	0.0034 (8)	0.0031 (7)	0.0002 (7)
C3A	0.0255 (8)	0.0299 (8)	0.0249 (7)	0.0042 (7)	0.0017 (7)	0.0012 (7)
C4	0.0310 (9)	0.0268 (8)	0.0324 (8)	0.0032 (8)	−0.0011 (7)	0.0029 (7)
C5	0.0293 (9)	0.0276 (8)	0.0363 (9)	−0.0007 (8)	−0.0049 (7)	0.0014 (7)
C5A	0.0253 (8)	0.0300 (8)	0.0311 (8)	0.0026 (7)	−0.0042 (7)	0.0048 (7)
C6	0.0260 (9)	0.0436 (11)	0.0450 (10)	0.0037 (8)	0.0069 (8)	0.0072 (9)
C7	0.0337 (10)	0.0403 (10)	0.0391 (10)	0.0121 (9)	0.0021 (8)	−0.0002 (8)
C8	0.0370 (9)	0.0315 (8)	0.0277 (8)	0.0087 (8)	−0.0050 (7)	0.0024 (7)
C8A	0.0284 (8)	0.0252 (7)	0.0224 (7)	0.0017 (7)	−0.0006 (6)	0.0031 (6)
C8B	0.0234 (8)	0.0284 (8)	0.0238 (7)	0.0000 (7)	0.0024 (6)	0.0013 (6)
C9	0.0369 (10)	0.0447 (10)	0.0341 (9)	0.0101 (9)	−0.0045 (8)	0.0023 (8)
C10	0.0452 (11)	0.0324 (9)	0.0361 (9)	−0.0010 (9)	−0.0088 (8)	−0.0034 (7)
C11	0.0363 (10)	0.0347 (10)	0.0607 (13)	−0.0060 (9)	−0.0088 (10)	0.0054 (9)
C12	0.0527 (12)	0.0303 (9)	0.0373 (9)	0.0080 (9)	−0.0072 (9)	−0.0012 (8)
O1	0.0274 (6)	0.0322 (6)	0.0310 (6)	−0.0012 (5)	−0.0001 (5)	−0.0018 (5)
O2	0.0287 (7)	0.0547 (9)	0.0530 (8)	−0.0036 (7)	−0.0043 (6)	−0.0080 (7)
O3	0.0599 (10)	0.0579 (9)	0.0414 (8)	0.0095 (9)	0.0002 (7)	−0.0130 (7)

### Geometric parameters (Å, °)

**Table:**

C2—O2	1.202 (2)	C6—H6B	0.9700
C2—O1	1.360 (2)	C7—C8	1.330 (3)
C2—C3	1.497 (3)	C7—H7	0.9300
C3—C9	1.319 (3)	C8—C12	1.499 (3)
C3—C3A	1.497 (2)	C8—C8A	1.513 (2)
C3A—C4	1.515 (2)	C8A—C8B	1.512 (2)
C3A—C8B	1.519 (2)	C8A—H8A	0.9800
C3A—H3	0.9800	C8B—O1	1.459 (2)
C4—C5	1.535 (2)	C8B—H8B	0.9800
C4—H4A	0.9700	C9—H9A	0.9300
C4—H4B	0.9700	C9—H9B	0.9300
C5—C10	1.533 (3)	C10—O3	1.191 (2)
C5—C11	1.538 (3)	C10—H10	0.9300
C5—C5A	1.552 (2)	C11—H11A	0.9600
C5A—C6	1.544 (3)	C11—H11B	0.9600
C5A—C8A	1.553 (2)	C11—H11C	0.9600
C5A—H5A	0.9800	C12—H12A	0.9600
C6—C7	1.504 (3)	C12—H12B	0.9600
C6—H6A	0.9700	C12—H12C	0.9600
			
O2—C2—O1	121.68 (18)	C8—C7—H7	123.8
O2—C2—C3	129.07 (18)	C6—C7—H7	123.8
O1—C2—C3	109.23 (15)	C7—C8—C12	128.02 (18)
C9—C3—C2	123.43 (17)	C7—C8—C8A	109.96 (16)
C9—C3—C3A	131.43 (18)	C12—C8—C8A	122.00 (16)
C2—C3—C3A	105.12 (14)	C8B—C8A—C8	112.67 (13)
C3—C3A—C4	123.15 (14)	C8B—C8A—C5A	106.51 (13)
C3—C3A—C8B	100.87 (14)	C8—C8A—C5A	101.81 (14)
C4—C3A—C8B	109.53 (13)	C8B—C8A—H8A	111.8
C3—C3A—H3	107.4	C8—C8A—H8A	111.8
C4—C3A—H3	107.4	C5A—C8A—H8A	111.8
C8B—C3A—H3	107.4	O1—C8B—C8A	114.69 (13)
C3A—C4—C5	108.77 (14)	O1—C8B—C3A	104.61 (13)
C3A—C4—H4A	109.9	C8A—C8B—C3A	111.88 (13)
C5—C4—H4A	109.9	O1—C8B—H8B	108.5
C3A—C4—H4B	109.9	C8A—C8B—H8B	108.5
C5—C4—H4B	109.9	C3A—C8B—H8B	108.5
H4A—C4—H4B	108.3	C3—C9—H9A	120.0
C10—C5—C4	109.89 (16)	C3—C9—H9B	120.0
C10—C5—C11	104.81 (16)	H9A—C9—H9B	120.0
C4—C5—C11	110.74 (15)	O3—C10—C5	126.73 (19)
C10—C5—C5A	106.41 (14)	O3—C10—H10	116.6
C4—C5—C5A	114.30 (15)	C5—C10—H10	116.6
C11—C5—C5A	110.18 (15)	C5—C11—H11A	109.5
C6—C5A—C5	118.22 (15)	C5—C11—H11B	109.5
C6—C5A—C8A	102.79 (14)	H11A—C11—H11B	109.5
C5—C5A—C8A	116.89 (14)	C5—C11—H11C	109.5
C6—C5A—H5A	106.0	H11A—C11—H11C	109.5
C5—C5A—H5A	106.0	H11B—C11—H11C	109.5
C8A—C5A—H5A	106.0	C8—C12—H12A	109.5
C7—C6—C5A	101.51 (16)	C8—C12—H12B	109.5
C7—C6—H6A	111.5	H12A—C12—H12B	109.5
C5A—C6—H6A	111.5	C8—C12—H12C	109.5
C7—C6—H6B	111.5	H12A—C12—H12C	109.5
C5A—C6—H6B	111.5	H12B—C12—H12C	109.5
H6A—C6—H6B	109.3	C2—O1—C8B	108.23 (13)
C8—C7—C6	112.41 (18)		
